# Effect of the Elastomer Matrix on Thermoplastic Elastomer-Based Strain Sensor Fiber Composites

**DOI:** 10.3390/s20082399

**Published:** 2020-04-23

**Authors:** Antonia Georgopoulou, Claudia Kummerlöwe, Frank Clemens

**Affiliations:** 1 Department of Functional Materials, Empa—Swiss Federal Laboratories for Materials Science and Technology, Überlandstrasse 129, 8600 Dübendorf, Switzerland; 2 Department of Mechanical Engineering (MECH), Vrije Universiteit Brussel (VUB), and Flanders Make Pleinlaan 2, B-1050 Brussels, Belgium; 3 Faculty of Engineering and Computer Science, Osnabrück University of Applied Sciences, 49076 Osnabrück, Germany; c.kummerloewe@hs-osnabrueck.de

**Keywords:** fiber composites, piezoresistive response, strain sensor, electro-mechanical testing

## Abstract

In this study, a thermoplastic elastomer sensor fiber was embedded in an elastomer matrix. The effect of the matrix material on the sensor properties and the piezoresistive behavior of the single fiber-matrix composite system was investigated. For all composites, cycling test (dynamic test) and the relaxation behavior at different strains (quasi-static test) were investigated. In all cases, dynamic properties and quasi-static significantly changed after embedding, compared to the pure fiber. The composite with the silicone elastomer PDMS (Polydimethylsiloxane) as matrix material exhibited deviation from linear response of the resistivity at low strains and proved an unsuitable choice compared to natural rubber. The addition of a spring construct in the embedded sensor fiber natural rubber composite improved the linearity at low strains but increased the mechanical and electrical hysteresis of the soft matter sensor composite. Using pre-vulcanized natural rubber improved linearity at low strains and reduced significantly the stress and relative resistance relaxation as well as the resistance hysteresis, especially if the resistance remained low. In both cases of the pre-vulcanized rubber and the spring structure, the piezoresistive behavior was improved, and at the same time, the stiffness of the system was increased indicating that using a stiffer matrix can be a strategy for improving the sensor properties.

## 1. Introduction

The recent progress in the field of conductive composite materials has produced many attractive new opportunities for sensor development. Inspired by the commonly used metal strain gauges that offer repeatable, precise strain measurements at low strains [1] but have a high Young’s modulus, researchers have developed elastic sensors based on polymers and their composites. Rubber-based strain sensors can easily measure in a twentyfold range and have already found application in health monitoring [2], robot motion detection [3,4] and human interface devices [5,6]. For a wide range of applications, it is of great interest to study the embedding of soft sensor systems in a polymer or composite matrixes, as this is often the goal in the creation of stretchable electronics or soft robotic systems [7]. This method often involves the combination of a conductive phase inside a polymer matrix that is not conductive [8].

Elastomer-based piezoresistive sensors can be manufactured by combining a conductive filler and an elastomer matrix. Under strain, the resistivity of the composite changes due to geometrical changes [9,10], changes in the bandgap of interatomic spacing [11] and the electron tunneling effect [12]. However, piezoresistive rubber sensors usually suffer from high hysteresis and time-dependent resistance change [13], which limits their precision. Many encouraging attempts have proven that conductive elastomers have attractive properties and can be used for the development of soft strain sensing systems [14,15,16]. In this attempt, a piezoresistive thermoplastic elastomer-based strain gauge fiber was developed [6]. This fiber sensor, with a diameter of 0.3 mm, was based on the thermoplastic elastomer styrene–ethylene/butylene–styrene triblock copolymer (TPS) filled with a high loading of non-spherical carbon black to achieve a relatively linear resistance change over a strain range up to 200% [17]. This approach has several advantages such as low material cost (TPS and carbon black), simple manufacturing, applicability to a wide range of geometries (if adhered to sample and embedded in a matrix) and low stiffness [18,19]. Due to the high filling level significantly above the percolation threshold, a stable electrical response with low hysteresis and relaxation could be achieved [20].

Soft condensed sensor fibers have been applied on the surface of soft elastic structures like textiles and silicone structures [7,19]. The small diameter of the fiber makes the handling difficult for many applications, and the approach of attaching them to a substrate is not enough to ensure robustness and easy handling. Alternatively, the fiber sensors could be embedded inside the material, and in that way the sensor can be protected from environmental effects and harsh handling conditions. Abshirini et al. produced a PDMS (polydimethylsiloxane)/Carbon nanotubes sensor where the conductive filler was embedded in the elastomer matrix, and their results showed a linear electrical response and good mechanical behavior for the system after being embedded [21]. However, choosing the right matrix material can be important to avoid dissociation or diffusion of the conductive phase within the matrix but also unwanted reactions at the interfaces [22]. Often, it is of great importance to investigate the properties of the embedded sensor and compare them with the properties of the sensing element before embedding [23], but there are not a lot of studies in the field that examine how the piezoresistive response of the sensor changes after the embedding.

Silicone elastomers can be a good option for a flexible substrate in sensor development [24], and they have been used in the past in embedding sensitive structures like sensors [25]. Natural rubber is a soft material, with high tensile strength among rubbery materials that can undergo very large deformations [26]. It is known for its good resilience, ease of manufacture and high fatigue resistance [27,28]. Natural rubber is susceptible to stress relaxation phenomena that are a result of physical and chemical effects which can be reduced with the appropriate material design and compounding [27]. When natural rubber is cross-linked with peroxides, it exhibits improved mechanical properties and low hysteresis and creep [29]. Natural rubber materials have been used as a matrix material for strain sensor applications [30] and are a very suitable option for soft sensor applications. 

Because the matrix effect on piezoresistive sensor properties of embedded elastomer-based strain sensor fibers has not been reported so far, PDMS and natural rubber were examined as matrix materials. To examine the effect of the matrix on the piezoresistive behavior properly, single sensor fibers and composites were investigated by electromechanical analysis. To compare the different configurations, the dynamic response and relaxation effect for both, the mechanical stress and the electrical resistance during tensile testing were investigated. Thus, a reliable and robust sensor system that can be used in relevant applications can be created by selecting the right matrix material. 

## 2. Materials and Methods

### 2.1. Sensor Fiber Composite Preparation

The production process of the piezoresistive sensor fiber was published in previous work [1]. A styrene–ethylene/butylene–styrene triblock copolymer (TPS), (KRAIBURG TPE GmbH and Co KG, Waldkraiburg, Germany) was mixed with 50 vol% Ensaco 250 carbon black (CB) (TIMCAL Graphite and Carbon, Bodio, Switzerland) in an internal mixer (HAAKE Polylab OS, Thermo Electron Corporation, Karlsruhe, Germany). The material was then extruded into fiber with a diameter of 0.3 mm using a capillary rheometer (Rosand RH7, Malvern Panalytical GmbH, Herrenberg, Germany [1,2]. 

The pre-vulcanized natural rubber was prepared by adding 1 phr tert-butylhydroxyperoxide (Sigma Aldrich, St. Louis, MO, USA) and hydroxyacetone (Sigma Aldrich, St. Louis, MO, USA) to natural rubber latex. The peroxide has a decomposition temperature of 70 °C and is not water-soluble. Before casting, the liquid natural rubber and the crosslinking agent were placed on a heat plate set to 70 °C and mixed for 10 minutes at 170 rpm to pre-vulcanize the material before casting.

For the sensor composites, the piezoresistive fiber was cast with the silicone elastomer DC3140, supplied by Dow Corning, and natural rubber latex (centrifuged natural latex, 62% solids) supplied by Holden’s Latex. After the casting, the samples were left to dry overnight at room temperature. The molds used for casting had a dimension of 70 × 10 × 2 mm and were produced using an FDM 3D printer (K8200 3D printer, Velleman NV, Gavere, Belgium). 

The spring structure was designed in Autodesk Inventor, sliced with Simplify3D and printed with the Velleman K8200 FDM printer using a 0.4 mm nozzle and PLA (polylactic acid) filament. Since PLA was used for the spring structure, it was important that maximum strain within the spring not exceed the yield point of 2% [31,32]. The developed spring consisted of meandering half-circles with a diameter and thickness of 2.3 mm and 0.65 mm, respectively. The center-to-center distance was 4 mm to introduce a small recurve that eased the expansion of the spring. At 20% elongation, FEM simulation indicated that the maximum internal strain was 1.8% and therefore below the expected yield elongation. 

**Figure 1:**
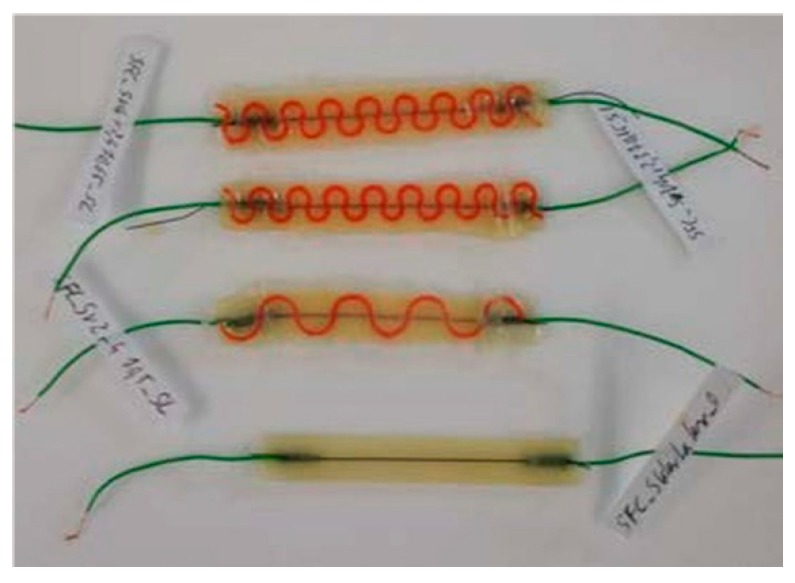
Different embedded sensor systems with (top) and without (bottom) the embedded support spring structure.

Before the casting step, piezoresistive fiber samples were pre-stretched to 50% elongation, held for 60 seconds and allowed to relax for at least two hours before the casting processing steps. After pre-stretching the fibers, copper wires were fixed on both fiber ends by an aluminum crimp. The connected fiber was placed inside a 3D printed mold and fixed with adhesive tape. For the sample with the spring structure, the spring was placed inside the mold before placing the fiber. Then the respective liquid elastomer was cast onto the fiber and cured after the embedding. Figure 1 shows selected examples of embedded sensor fibers with and without the spring structure.

### 2.2. Tensile Testing

**Figure 2:**
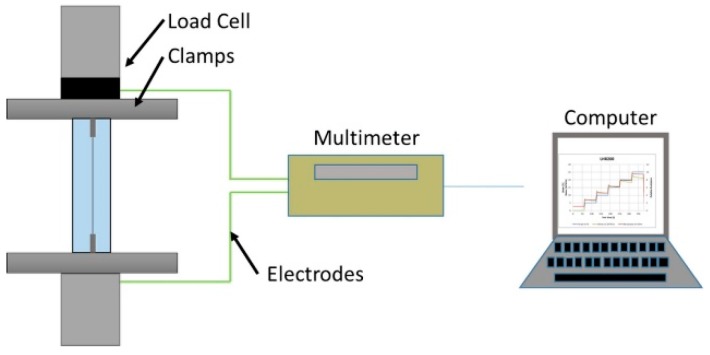
Tensile test setup with pneumatic grips and multimeter probes.

Tensile tests were performed on a Zwick Roell Z005 (Zwick GmbH, Ulm, Germany) tensile testing machine with a 200 N load cell. A Keithley 2700 multimeter was used to measure the electrical resistance in situ. The different embedded sensor systems were clamped with Zwick pneumatic 100 N grips (4 bar air pressure) and connected to the multimeter (Figure 2). For the single fiber tests, standard monofilament testing frames with a 50 mm gauge length were used [33]. For all the tensile tests, the force was measured while the strain was changing. The change in strain was determined by the displacement in the length of the sample.

For the quasi-static tensile measurements, the composites were pre-loaded with 0.1 N and held in position for 60 seconds. The samples were elongated at a speed of 50 mm/min to 5%, 10%, 15%, 20% and 25% of strain. At each strain stage, the sample was statically held for 60 seconds. The pre-force was not applied for tests on the single fibers.

For the dynamic tensile measurements, the composites were pre-loaded with a force of 0.1 N and held in position for 60 seconds (similar to quasi-static measurements). Later the samples were elongated and retracted 20 times between 0% and 20% strain with a speed of 50 mm/min. The first and last cycle were investigated. Similar to quasi-static measurements, single fibers were not pre-loaded. To calculate the mechanical stress, the cross-sections of the fibers were measured with an optical microscope. In the case of the fiber embedded in an elastomer matrix, the dimensions of the mold were used. The relative resistance was calculated by the following equation:

(1) $$R_{\mathrm{rel}}=\frac{R-Ro}{Ro}$$

The sensitivity of strain gauges is commonly described with the gauge factor (GF) in Equation (2),

(2) $$GF=\frac{\frac{R-R_{0}}{R_{0}}}{\epsilon }$$

where R is the resistance at strain ϵ, and $R_{0}$ is the initial resistance.

The values of the initial resistance R_0_ for the sensors systems can be found in Table 1.

The mechanical and electrical drift during the dynamic testing were calculated by finding the percentage difference between the value for the stress and the resistance, respectively, at the peak (max) of the first cycle from the value at the peak of the last cycle. The same was done by calculating the values of the drift for the minimum points (min) at the transition between the first and second cycle and at the end of the last. The hysteresis at a specific strain was calculated as the percentage difference between the value for the stress or the relative resistance at the same strain during loading and unloading. 

## 3. Results and Discussion

### 3.1. Dynamic Testing

**Figure 3:**
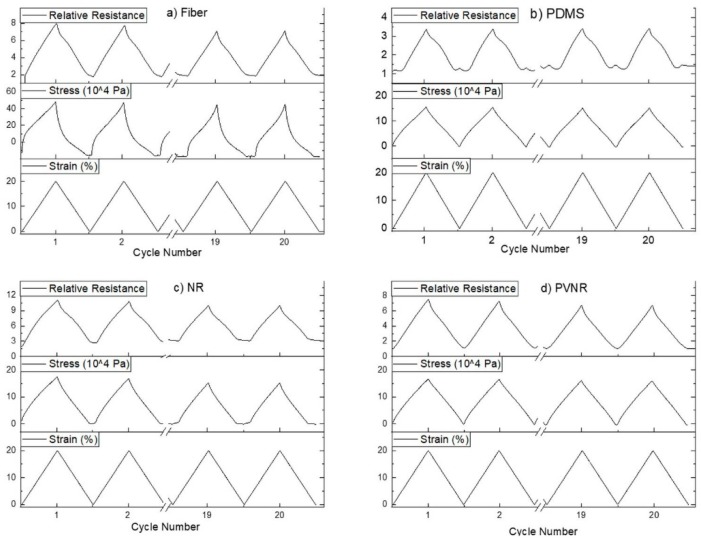
First and last two cycles of 20 cyclic stretches from 0% to 20% for (**a**) the single fiber and the sensor fiber embedded in (**b**) PDMS, (**c**) natural rubber (NR) and (**d**) pre-vulcanized natural rubber (PVNR).

In an attempt to assess the reliability and reproducibility of the sensors embedded in the elastomer matrix, a cyclical straining of 20 cycles from 0% to 20% strain was done for different elastomer matrix materials. Results of stress and resistance measurements are shown in Figure 3. 

For the case of the single fiber (Figure 3a), the results revealed that the sensor fiber exhibited good sensitivity and repeatability of the change in resistance under dynamic mechanical testing. However, at low strain a plateau in the mechanical stress and the electrical resistance could be observed. This loss of linearity at low strains could affect the performance of the sensor at low strains and could be linked with the relaxation phenomena of the thermoplastic elastomer such as the TPS that was used for the sensor fiber. Calculation of the signal drift for the peaks of maximum strain showed that there was a downwards drift for the relative resistance, which was calculated at 11.9%, and this could be undesirable for many applications where accuracy overtime is required. 

In an attempt to improve the properties of the sensor fiber and at the same time protect the delicate, very thin fiber from environmental effects and wrong handling that could damage the fiber, the sensor was embedded in different matrix materials. The dynamic loading and unloading test was repeated for the embedded sensor system in order to investigate how the sensor behavior changes after the embedding. Silicone elastomers like PDMS have attracted great interest and are used quite often in many applications such as stretchable electronics and devices [34]. For the sensor fiber embedded in PDMS (Figure 3b), it was seen that the system had good repeatability for the stress and the electrical resistance. However, a lagging phase was observed at the beginning of the loading, while as the stain and stress increased, the relative resistance remained almost stable and then started to increase following the change in strain. This behavior has been observed in elastomers before and it has been suggested that it is linked with the changes in the conductive network and the viscoelastic behavior of the elastomer [35]. At the end of the loading and the transition between two consequent cycles, a secondary small peak was observed. This secondary peak at low strains could have occurred by the interactions at the interface between the piezoresistive fiber and the elastomer matrix or a possible buckling effect due to shrinkage of the elastomer during the curing process. Zhang et al. also found a secondary peak in their piezoresistive strain sensors made of thermoplastic polyurethanes and carbon nanotubes and explained the effect with competing conductive network formation and breaking processes [36]. As for the drift of the relative resistance, in this case, it was much reduced to 1.3% for the maximum strain in the cycle, showing that embedding the fiber can improve the sensor properties, but the loss of linearity at low strains and the appearance of a secondary peak make this composite sensor system an unattractive option for use at low strains (below 5%).

Another elastomer material that was chosen as a candidate matrix material was natural rubber in two different forms: non-pre-vulcanized and pre-vulcanized with peroxide. The test for the composite system with the non-vulcanized natural rubber showed a good correlation between the resistance and the strain, but the plateau that was observed in the unloading for the single fiber was also present in the case of the natural rubber composite system. A secondary peak in piezoresistive sensor composites prepared with natural rubber was not observed, and the plateau width was similar to that of the single sensor fiber. The signal drift from the beginning to the end of the cycles was calculated at 10.2%, which was lower than in the case of the single fiber. 

In the case of pre-vulcanized rubber, the cyclical testing revealed good piezoresistive behavior and reliability, but in this case, the linearity of the relative resistance at both loading and unloading was very good even at low strains. The phase shift that appeared in the case of the PDMS was not present in this system, and the plateau and bad linearity that was present in the other systems did not appear to make the pre-vulcanized natural rubber as a matrix material a good option for a reliable sensor, even at low strains, for use in long-term applications. 

**Figure 4:**
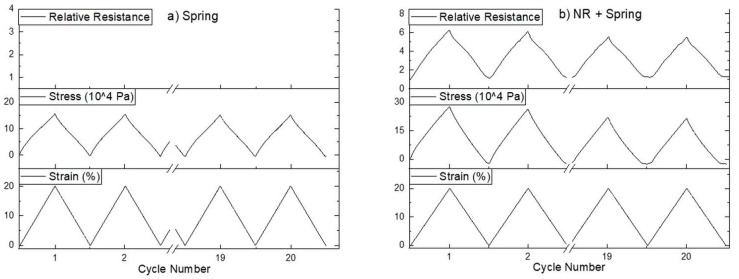
Twenty cyclic stretches from 0% to 20% for (**a**) the 3D printed spring structure and (**b**) the piezoresistive composite with natural rubber as casting material and the 3D printed spring of which the first and last two cycles are shown.

Elastomers are known for showing a relaxation behavior at higher strains. By implementing a 2D spring structure in the embedded fiber sensor, the purpose is to increase the elastic range of the composite to reduce the effect of the relaxation in the piezoresistive behavior. The PLA spring structure was produced by 3D printing; its mechanical behavior was assessed by cyclical tensile testing on a single spring structure (Figure 4a), and the response was symmetric and repeatable. 

After testing the pure PLA spring structure, the spring was embedded in the sensor fiber composite with the natural rubber as a matrix material. The dynamic test showed that the plateau at low strains was reduced compared with the case of the single fiber or the natural rubber without the spring structure. The signal drift for this system was 11.4% for the peaks from the first to the last, a similar trend as observed in the rest of the systems.

**Figure 5:**
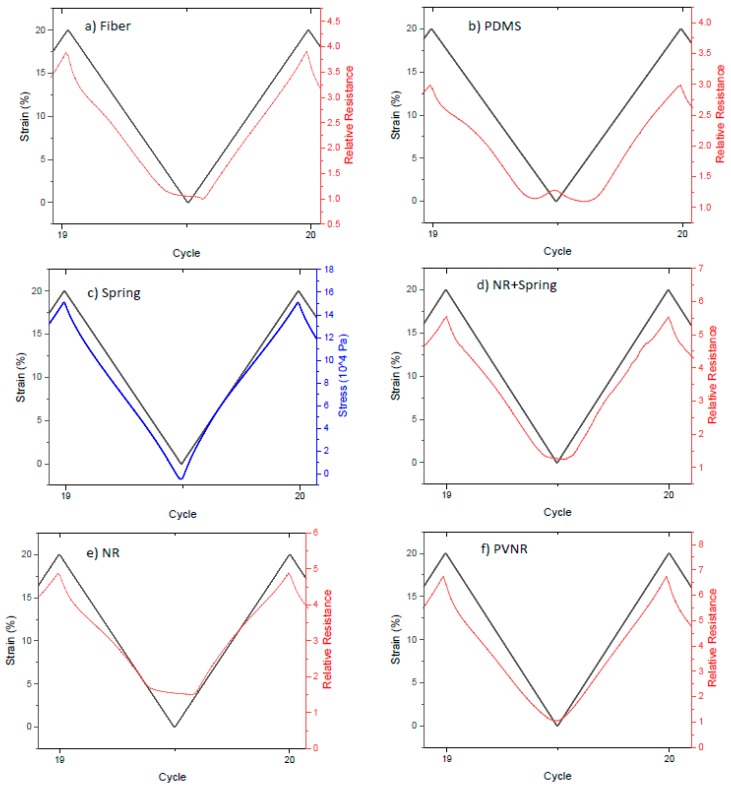
Overview of the electrical resistance (**a,b,d,e,f**) for the fiber sensor composites and the mechanical response of the 3D printed spring structure (**c**) between the 19th and 20th cycle of the cyclic tensile test.

Figure 5 summarizes the electrical resistance behavior during mechanical strain for the different sensor fiber composites. By embedding the piezoresistive sensor fiber in the natural rubber and in the natural rubber matrix with an integrated spring, at low strains a plateau could be observed, making the sensor a bad option for applications at low strains. Embedding the TPS-based sensor fiber into a pre-vulcanized natural rubber matrix significantly changed the piezoresistive properties of the resulting sensor composites. The effect of the matrix material can be considered important for all relevant applications such as wearable stretchable devices and soft robotic systems. 

**Figure 6:**
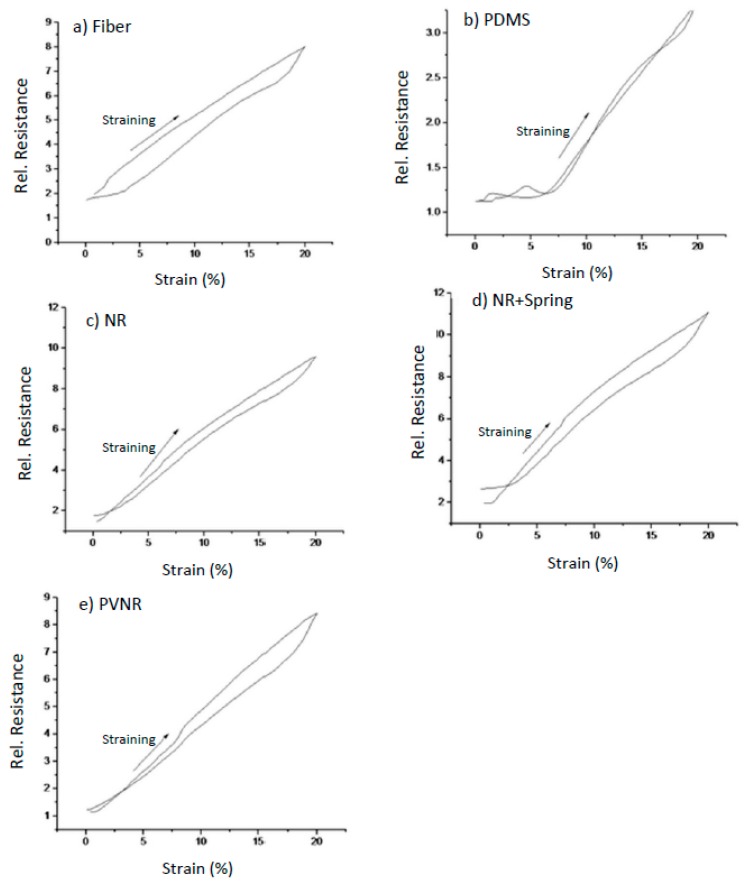
The relative resistance as a function of the strain during the first cycle of the dynamic testing for (**a**) the single fiber and the fiber embedded in (**b**) PDMS, (**c**) natural rubber (**d**), natural rubber with the spring structure and (**e**) pre-vulcanized natural rubber.

Dynamic cyclical testing can provide useful insights about the mechanical and electrical hysteresis in the different systems. Important conclusions about how the hysteresis affects the piezoresistive behavior of the system can be drawn by comparing the values for the stress and the resistance at the same strain during loading and unloading. The electrical hysteresis was calculated for the different systems for a 10% strain based on the first cycle of the dynamic testing (Figure 6).

In the case of the pure fiber, the electrical hysteresis was the highest of all other systems (Table 2), proving that embedding the fiber in an elastomer matrix can reduce the hysteresis of the relative resistance and improve the behavior of the sensor. 

For the case of the PDMS, the hysteresis was the lowest, and the lagging phase and loss of linearity at low strains was visible, where even though the strain increased, the relevant resistance remained constant. For the mechanical stress, the hysteresis increased in all embedded systems compared to the single fiber, except for the natural rubber. The electrical hysteresis was significantly lower for the embedded systems except for the composite sensor with the spring structure. The case of the spring stood out, as the hysteresis was four times higher than the rest of the systems and the pure fiber. It is assumed that this high hysteresis occurred because of the spring structure design. The structure was designed for a 20% strain.

**Figure 7:**
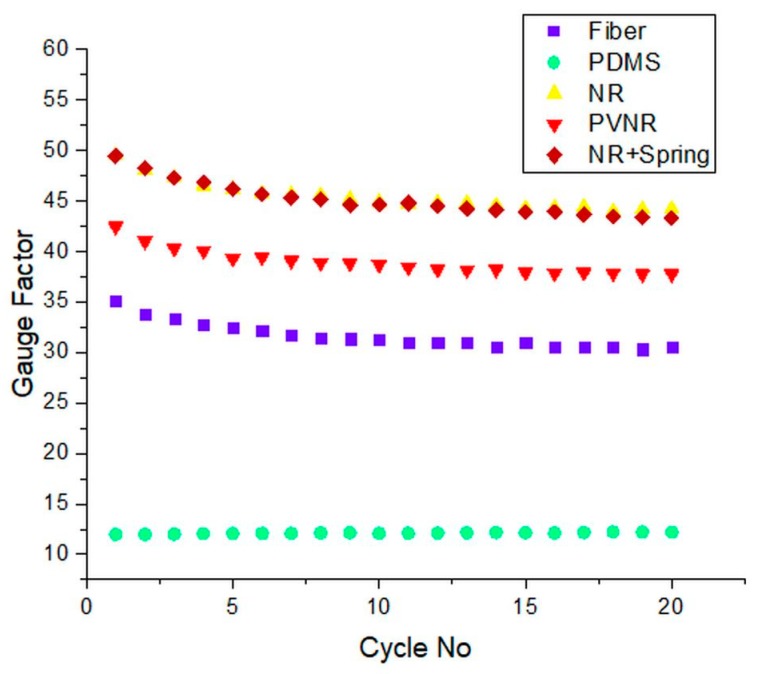
The gauge factor calculated for the single fiber and the different fiber embedded sensor materials during cyclical testing.

The gauge factor is often used as a measure of the sensitivity of a sensor. The gauge factor was calculated for the different systems during the cyclical testing (Figure 7). 

The gauge factor followed a downwards drift for the first cycles and stabilized after five cycles around the same value. The natural rubber and natural rubber with the spring had the highest sensitivity, followed by the pre-vulcanized natural rubber, showing that embedding the sensor fibers in elastomer matrixes can improve the sensitivity. On the other hand, the sensor system with PDMS as matrix material had the lowest gauge factor, which was 80% lower than that of the single fiber. However, the gauge factor for this composite was very constant over the cycle numbers. Embedding sensor fibers in composite systems do not always have a beneficial effect on its properties. For example, in this case, the wrong elastomer (PDMS) was chosen, because even though it had the lowest relaxation, it added a lagging phase and reduced the sensitivity of the system. On the other hand, the pre-vulcanized natural rubber improved the linearity at low strains and at the same time improved the sensitivity and decreased the hysteresis during dynamic cyclical loading. 

### 3.2. Quasi-Static Test and the Effect of Relaxation

**Figure 8:**
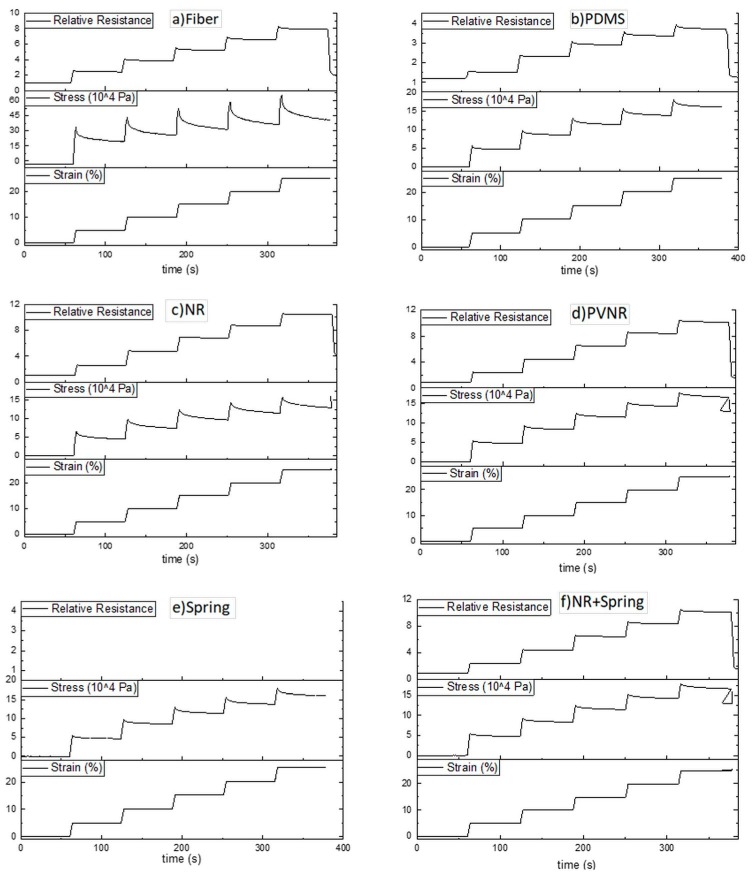
Quasi-static stepwise tensile test for (**a**) the single fiber, the fiber embedded in the elastomer (**b**) PDMS, (**c**) natural rubber, (**d**) pre-vulcanized natural rubber, (**e**) the spring structure and (**f**) the fiber embedded in natural rubber with the spring structure.

Elastomers such as the TPS of the sensor fiber and also the elastomers used as matrix materials are linked with stress relaxation effects that also affect the output signal of the sensor. In order to assess the effect of the relaxation on the piezoresistive behavior of the sensor, a quasi-static tensile test was performed, where for six steps the strain was held constant for 60 seconds. In every step, the strain was increased by 5%. The quasi-static test was performed for the fiber and the composite, in which the fiber sensor was embedded in the different elastomers (Figure 8). 

Figure 8a shows the results of the change in relative resistance during the tensile test and a stepwise increase of strain for the sensor fiber. During the strain phases, the piezoresistive fiber showed a steady increase in resistance and stress. When the strain was held constant, the stress decreased about 42% in each hold stage due to stress relaxation, and the resistance decreased by approximately 9% due to the high filling level and geometry of the carbon black [20]. The stress relaxation in every step decreased at higher strains, and a similar trend was observed for the case of the relative resistance. The relaxation behavior in elastomers was affected by the molecular structure of the elastomer, which may have important implications for time-dependent applications. Therefore, embedding the sensor in an elastomer matrix may affect the relaxation behavior. The different values for the mechanical and electrical relaxation for the quasi-static stepwise were calculated for the fiber and all the different composites (Table 3).

In the case of the PDMS (Figure 8b) the stress relaxation was reduced to 15%, and the electrical relaxation was also approximately half of that in the case of the pure fiber. The natural rubber (see Figure 8) did not reduce the mechanical relaxation as much as the PDMS did, but it was lower in comparison to the single fiber. The implementation of a 2D spring structure would increase the elastic behavior of the composite and as a result affect time-dependent behaviors, as was seen during the dynamic testing, reducing the relaxation of the composite system. The PLA spring structure was produced by 3D printing, and its mechanical behavior was assessed by tensile testing on a single spring structure. Even though stress relaxation behavior was observed during a quasi-static test (Figure 8e), the mechanical and electrical relaxation for the natural rubber with the spring structure was lower than the system with the natural rubber at low strains. At high strains, closer to the breaking point of the spring structure, the relaxation was higher for the system with the spring compared to the one without the spring. The sensor composite with pre-vulcanized rubber as matrix material had the lowest stress relaxation than any of the other systems at low and at high strains. The electrical relaxation was similar to the system with non-vulcanized natural rubber. Melnykowycz et al. investigated different elastomer sensor materials using combined quasi-static and dynamic tensile tests [17]. No correlation between mechanical and electrical relaxation was observed in this study. The reason for the lower mechanical relaxation was the pre-vulcanization of the natural rubber [37].

During the quasi-static test, it became evident for all the systems that the relaxation for both the stress and the resistance was reduced at higher strains. All the sensor fiber composites had lower relaxation properties compared to the single fiber, proving that embedding the sensor fibers in elastomer polymer matrixes can be a good strategy for reducing both the mechanical and the electrical relaxation. In the case of the composites, the behavior was dominated by the elastomer matrix that had lower relaxation than the TPS elastomer that was responsible for the relaxation in the case of the single fiber. The gauge factor in both the cases of high and low strains was calculated after the relaxation, and it was seen that as the relaxation was lower at high strains, the gauge factor was lower at high strains. The gauge factor decreased in the case of the PDMS but increased in the rest of the cases, showing that embedding sensors in elastomer matrixes can have a positive but also a negative effect on the sensitivity. For the other cases, the gauge factor was higher than the factor for a single fiber, showing that embedding the fibers can have a beneficial effect on the sensitivity of the composite sensor. The system with the spring and the fiber embedded in the pre-vulcanized natural rubber had the highest sensitivity compared with the other systems.

### 3.3. Calculation of Stiffness 

**Figure 9:**
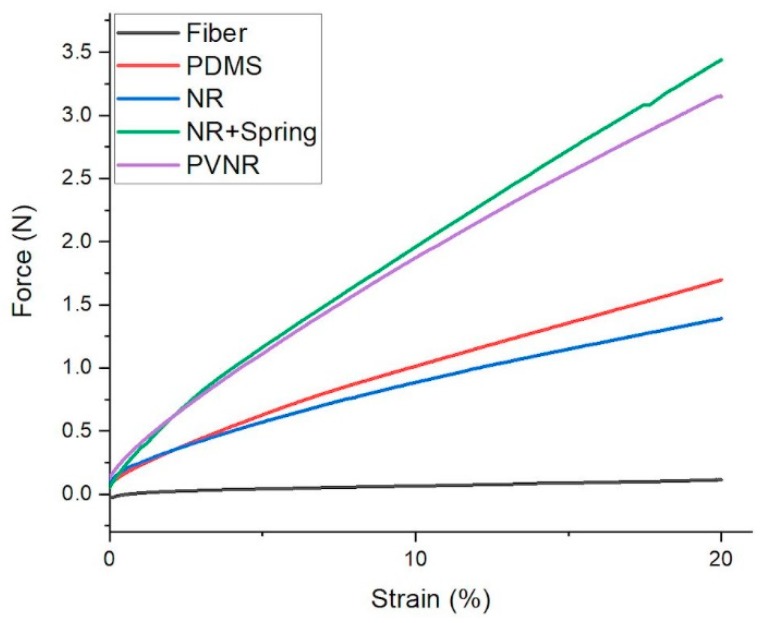
Tensile force vs. strain behavior of single piezoresistive (grey solid line) fiber and different fiber embedded sensor materials between 0 % and 20 % during the first cycle of the cyclic tensile test.

To investigate the mechanical behavior of the samples, the tensile force was plotted versus the strain for the first cycle in the dynamic test measurement. The mechanical properties of a single piezoresistive TPS/CB fiber, and embedded the fiber into different matrices resulting in sensor fiber composite structures, are shown in Figure 9.

It can be seen in Figure 9 that the single fiber did not have a noticeable force due to its small cross-section. Embedding the sensor fiber in non-vulcanized NR and DC3140 silicone led to an increase in cross-section and therefore also an increased required force of 1.2 N to 1.7 N to achieve 20% strain. For a pre-vulcanized and spring structure embedded in natural rubber fiber composites, the required force increased further to 3.2 N and 3.5 N, respectively. The stiffness of the pre-vulcanized natural rubber matrix was about 70 % higher in comparison with the natural rubber (25 × 10^4^ compared with 15 × 10^4^ Pa for the natural rubber system). The higher stiffness of pre-vulcanized natural rubber is a strong indicator of successful crosslinking. Additionally, adding the spring structure increased the stiffness as expected. It can be concluded that by using a stiffer matrix, the sensor properties of the embedded fiber composite can be improved, especially at lower strains.

## 4. Conclusions

In this attempt, the strategy of embedding a sensor fiber in elastomer matrix material in order to use it for soft robotic applications was explored. The electrical and mechanical characterization of different sensor fiber composites aimed to define the influence of the matrix on the electrical sensor properties. From the comparison of two different matrixes, PDMS and natural rubber, we can conclude that the matrix material significantly affects the sensor performance of an embedded piezoresistive fiber in comparison to a single fiber. The effect of relaxation, which is linked with elastomer material and affects the piezoresistive behavior of the composite material, can be minimized by choosing the right matrix material. In all the cases of the embedded systems, the stress and resistance relaxation were reduced significantly, but in the case of the silicone elastomer and the natural rubber matrix during the cyclical tensile test, a plateau between consecutive cycles persisted. Especially in the case of the silicone elastomer, a secondary peak appeared in the plateau, which additionally reduced the reliability of the sensor at low strains. In order to remove this plateau, two different strategies were investigated successfully. In the first attempt, a PLA spring structure was 3D printed and embedded with the sensor inside the elastomer matrix. The addition of a spring structure to the natural rubber composite increased the elastic behavior of the system, and the plateau was reduced. However, the electrical and mechanical drift during dynamic testing increased compared to the other composite systems and even the single matrix. The other strategy was to use pre-vulcanized natural rubber as a matrix material. In this case, the plateau in electrical resistivity at low strain level could be removed. The stress relaxation was the lowest of all the other fiber composites tested in this attempt; the electrical and mechanical drift was lower, and the stiffness was similar in comparison to the composite with the embedded spring. Based on the results, we can assume that increasing the stiffness of the elastomer composite improves the dynamic properties of the sensor fiber composite, especially at lower strains.

Overall, the choice of the elastomer matrix material should be done carefully, as it can have unwanted effects on the sensor behavior and reliability of the system. Embedding sensor fibers in an elastomer matrix can lead to improved sensor properties and can lead to the creation of complex composite systems that can potentially be used in the field of wearable devices and soft robotics.

## Figures and Tables

**Table 1:** Initial resistance for the sensor systems. The values were used in this attempt to calculate the relative resistance. The systems include the sensor fiber and fiber composites with matrix of PDMS, NR (natural rubber), PVNR (pre-vulcanized natural rubber) and NR with an integrated spring structure.

Sensor	Initial Resistance (kΩ)
Single Fiber	10
PDMS	16
NR	4
PVNR	4
NR+Spring	4

**Table 2:** Electrical and mechanical properties during dynamic testing for the fiber and the different fiber embedded sensor systems.

	Dynamic Properties							
	Mechanical Drift			Electrical Drift			Gauge Factor	
	Max	Min	Hysteresis at 10%	Max	Min	Hysteresis at 10%	First Cycle	Last Cycle
Single Fiber	0.2%	6.4%	6.0%	11.9%	3.4%	15.5%	35.1	30.6
PDMS	4.1%	85.7%	8.6%	1.3%	22.3%	1.2%	15.0	12.2
NR	13.2%	72.0%	0.6%	10.2%	16.0%	1.4%	49.5	44.2
PVNR	4%	61.3%	11.2%	10.4%	8.8%	2.3%	42.3	37.6
NR+ Spring	24.3%	24.0%	50.2%	11.4%	8.4%	16.0%	49.5	43.3

**Table 3:** Electrical and mechanical properties during quasi-static test for the fiber and the different fiber embedded sensor systems.

	Quasi-Static Properties					
	Mechanical Relaxation %		Electrical Relaxation %		Gauge Factor	
	5% strain	20% strain	5% strain	20% strain	5% strain	20% strain
Single Fiber	42%	39%	9%	5.0%	32.7	29.4
PDMS	15%	11%	2.5%	4.2%	6.3	9.72
NR	28%	13%	4%	2.5%	32.9	38.6
PVNR	14%	7%	4%	2.0%	36.8	44.9
NR+Spring	26%	21%	3.5%	3.5%	36.6	42.2
